# Statistician, heal thyself: fighting statophobia at the source

**DOI:** 10.3389/fpsyg.2015.01558

**Published:** 2015-10-12

**Authors:** Aleksandar Aksentijevic

**Affiliations:** Department of Psychology, University of Roehampton, London, UK

**Keywords:** statistics anxiety, randomness, variance, probability, information

## Abstract

Notwithstanding the popularity of psychology courses throughout the world, educators face a constant and difficult problem of overcoming the fear of and dislike for statistics which represents one of the pillars of modern psychological science. Although the issue is complex and multifaceted, here I argue that “statophobia” might represent a rational and justified response to the sense of unease felt in contact with abstract statistical concepts which are often vague, circular or ill-defined. I illustrate the problem by briefly discussing two myths about the nature of probability and statistics, namely that probability and statistics generate knowledge and that the fault for not understanding probability lies solely with the subjective cognition which is incapable of comprehending deeper mathematical truth. I argue that the confident presentation of statistical methods hides numerous conceptual blind spots that students might be aware of and that need to be addressed before other causes of statistics anxiety can be tackled successfully.

## Who’s Afraid of the Big Bad… Central Limit Theorem?

Many candid persons, when confronted with the results of Probability, feel a strong sense of the uncertainty of the logical basis upon which it seems to rest. It is difficult to find an intelligible account of the meaning of “probability,” or of how we are ever to determine the probability of any particular proposition; and yet treatises on the subject profess to arrive at complicated results of the greatest precision and the most profound practical importance (Keynes, 1921, p. 56).

Teaching statistics represents every psychology lecturer’s baptism of fire. Facing a large auditorium packed with eager faces that start to sink into boredom and incomprehension as soon as the word “variance” is mentioned and its formula appears on the screen has filled many a new (as well as experienced) lecturer with a sense of foreboding and self-doubt. According to some estimates (e.g., Onwuegbuzie and Wilson, 2003), between 66 and 80% of students experience some degree of statistics anxiety.

Mathematics and statistics anxiety are related (Baloğlu, 1999) since statistics is formulated in the language of mathematics. Many of the causes of mathematics anxiety are transferrable to statistics, including difficulty of manipulating formulae as well as problems with performing arithmetical and algebraic operations. At the same time, research suggests that mathematics and statistics anxiety are distinct—if closely related—phenomena (Baloğlu, 2004). Some authors have observed utilization of different cognitive mechanisms (Cruise et al., 1985) and that statistical reasoning might be closer to verbal than mathematical reasoning (Buck, 1987). Like mathematics anxiety, statistics anxiety has been studied primarily using quantitative measures (e.g., STARS; Cruise et al., 1985). A number of dispositional and situational factors have been linked with statistics anxiety including gender, culture, tendency to procrastinate and reading ability (see Chew and Dillon, 2014a, for review).

Although some experts acknowledge the beneficial effects of medium anxiety levels (Keeley et al., 2008), statistics anxiety has been causally linked with reduced performance in a number of disciplines—from psychology (Lalonde and Gardner, 1993; Macher et al., 2011) and education (Onwuegbuzie et al., 2000) to business (Zanakis and Valenzi, 1997). Consequently, a number of “treatments” has been proposed including reducing mathematical content and the amount of hand calculation, keeping students engaged, using humor, increasing instructor confidence and immediacy (Chew and Dillon, 2014a) and teaching online (DeVaney, 2010).

What could be causing statistics anxiety? Since a number of researchers cite negative attitudes toward statistics as the cause (e.g., Watson et al., 2002; Chiesi and Primi, 2010), the question should be rephrased—what causes the negativity (in addition to the factors mentioned above)? Statistics can be distinguished from mathematics in one important way—it aims to “freeze,” quantify and package *uncertainty*—that fundamental imponderable of human existence. A recent systematic review of statistics anxiety literature (Chew and Dillon, 2014a) mentions only one study in which uncertainty features as a possible causal factor (Williams, 2013), and even there only as a psychological predisposition rather than an inherent property of statistics.

Although statistics teaching has come under increased scrutiny by the researchers, judging by the number of papers devoted to the topic in recent years, there is a creeping doubt that the problem lies not in the inability of students to “think properly” but in deep unresolved issues that underpin the foundations of probability and statistics. This is supported by the fact that expert researchers ostensibly exhibit an alarming lack of statistical aptitude—to the extent that the validity of most research findings in *most research fields* has been questioned (e.g., Ioannidis, 2005). Recurring episodes of heightened concern over statistical reasoning and performance of both students (current research topic) and experts (e.g., Cumming, 2014) suggest that the causes of anxiety and apprehension are at least partly to be found in the logic of statistical reasoning itself. Here, I briefly address two myths whose deconstruction might contribute to ameliorating the problem.

## Myth 1: Statistics Generates Knowledge

The development of powerful mathematical models and sophisticated inferential systems has engendered the belief that uncertainty is somehow controllable and—the worst of sins—conquerable. The ability to produce complex formulae which partition probabilities of various outcomes, weigh unequal conditional likelihoods and take into account prior knowledge does require mathematical sophistication that escapes many researchers, let alone students. At the same time, it fosters the mistaken impression that the formulae themselves generate qualitatively new information that is not present in the phenomena under observation.

If a pattern or a difference between objects is salient, our senses are sufficiently acute to detect and discriminate in most situations. Statistics becomes necessary when differences and dependencies become too small, numerous, complex, or remote to be analyzed by means of perception. This increase in informational distance between the observer and the phenomenon is managed via two basic steps. One is to exchange individual values/scores for a single number that hopefully retains maximum information. The second step is to quantify the uncertainty of this estimate. The amount of information conveyed by the mean is given by the variance. The more similar the scores, the lower the variance and the more informative the mean is. Here we face a paradox: The more informative the mean is, the less information there is in the population. To illustrate, in a population consisting of 4s, the mean of four conveys maximum information about the population. Yet, the population containing only 4s is maximally redundant and bereft of information (e.g., Shannon, 1948; Aksentijevic and Gibson, 2012). Thus, statistics provide most information about populations that possess no information at all. The more complex a data set, the less we can know about it. Rather than generating knowledge, statistics is at its best when no information is present.

The link between probabilistic models and real-life phenomena is tenuous at best. The use of probabilistic models in statistics is underpinned by a number of assumptions that can often not be confirmed empirically. Although this is dealt with by means of various methodological legerdemains, one example is sufficient to expose the students’ predicament. In order for results of statistical tests to be interpreted in terms of a particular statistical model (e.g., Gaussian), we must assume that the process in question is unchanging over time (i.e., ergodic; see Attneave, 1959). Given the dynamic, ever-changing nature of reality on all scales, it is difficult to understand how the assumption of ergodicity can be maintained. If students cannot articulate these concerns, there is no reason to believe that they are not aware of them. Perhaps, anxiety stems from inchoate understanding of the impossibility of reconciling the fundamental unknowability of most future outcomes and the apparent certainty with which laws of probability and statistical procedures are expounded by experts.

The apparatus of statistical reasoning has its origins in the inability of scientists to describe and predict outcomes of complex processes—either on macro (gambling; Hacking, 1975) or micro scales (molecular motion; Uffink, 2006). Rather than a major advance in the search for truth, statistics could justifiably be viewed as an admission of defeat in the face of phenomena that defy easy description. Probabilistic reasoning can be reduced to the following statement: *In the absence of information about the process under observation*, all outcomes are equally likely—anything can happen. This statement is easily converted into a mathematical expression and elaborated in a number of ways to account for different combinations of outcomes. Equally, a posteriori probabilities can be modified by additional information (Bayesian calculus). However, none of these operations produces new information in the sense of affecting the reality on the ground. Probabilistic reasoning is a posteriori by definition. The best it can do is to roughly *describe* certain processes that are inaccessible to unaided perception.

## Myth 2: It is all our Fault

An important contributory cause of statistics anxiety could be the constantly reinforced mantra, according to which human observers are failures at statistical reasoning (e.g., Kahneman and Tversky, 1972). This is in addition to apparent inability to reason logically (Wason, 1966) and well-documented biases observed in simplest perceptual tasks such as bisecting a line (e.g., Jewell and McCourt, 2000). According to the dominant paradigm, human mind, that supposedly unique natural system replete with ability and potential is at the same time highly fallible and incapable of understanding even the basic tenets of logic and probability. If we combine this with the reluctance to question and challenge the teacher (Cruise et al., 1985), is it surprising that students feel anxious and uncomfortable from the start? Statistics anxiety could be more pernicious than mathematics anxiety. Mathematics is an enclosed system which exists independently of observation (although its subjective origins should be acknowledged). By contrast, probability makes inferences about real-life phenomena which all of us deal with regularly and understand intuitively. When told that our intuitions about our own experience are wrong, we are more likely to doubt our overall competence.

One of the most difficult problems encountered by lecturers is explaining the concept of randomness. As confirmed by the massive literature devoted to the subject, students are not the only ones that have difficulties with it. When “randomly” assigning subjects to conditions or generating “random” patterns, they might find that the supposedly random process often generates patterns that appear regular and repetitive (Lopes, 1982). Soon, they might experience a cognitive dissonance between the given definitions of randomness and their own intuitions. For instance, a random process involves infinity and complete independence between outcomes (e.g., Falk, 1991). How are we supposed to interact with a process that produces completely unknowable outcomes? When asked to generate “random-like” sequences, students soon learn that their performance systematically departs from the “laws” of probability. Specifically, they are told that they produce too many alternations and too few streaks (Gilovich et al., 1985; Oskarsson et al., 2009). And yet, if the “correct” distribution is known in advance, the process cannot be random. Students’ anxiety might subside somewhat if they knew that mathematicians working for the RAND corporation were caught correcting tables of random numbers that were not sufficiently irregular (Gell-Mann, 1994).

The main source of confusion is the circular nature of “objective” probabilistic reasoning. Probabilistic models and ideas of randomness have subjective origins. Randomness represents abstract idealization of subjective complexity. Over time, it became so abstract as to lose any connection with its experiential sources. Randomization was invented in order to remove biases and preclude easy prediction. Randomization algorithms and other complex processes push the boundaries of complexity outside of the grasp of unaided perception and cognition. Is it then surprising that humans fail to understand randomness? Why would we expect humans whose cognition is pattern-based to be able to comprehend or generate sequences that lack any patterning or that conform to some probabilistic model? A random process can generate any outcome, leaving observers completely helpless. If they label a disordered sequence “random,” they are told that this is no more random than a sequence of zeros, forcing them to suppress their (correct) intuition which says that ordered patterns are more likely to be generated by a deterministic process and that random patterns are generated by complex processes which they cannot understand. Equally, if they characterize an ordered pattern as non-random, they are informed that they are wrong and that runs of identical symbols are often produced by random processes^1^.

Related to this, one of the most consistent (and anxiety-inducing) findings in psychology has been the observation that subjects perform poorly on tasks requiring partitioning and weighting probabilities in the presence of partial information (Keren, 1984; Mandel, 2008). A good example is the three-card problem which produces significant departures from probabilistic norm (Falk and Lann, 2008). There are thee cards—red/red, red/green and green/green. If a card is drawn that shows a red face, what is the probability that its other face is red? A majority of subjects (at least 65%) failed to give a correct answer (2/3), preferring the uniform partitioning of probabilities (1/2)^2^. Following similar results obtained in related experiments, the authors concluded that “The size of the deviations from truth caused by falsely applying uniformity might not be practically pernicious, nonetheless, such judgments are *wrong in principle*. (p. 331; italics mine)” This sounds like an admonishment of the imperfect mind for its inability to keep up with the eternal mathematical truth. Yet, probabilistic calculus emerged from subjective observation and deduction. Following mathematical elaboration and abstraction, it became too detached from experience to remain relevant to reasoning about every-day events—for which purpose it had been invented in the first place. How can intuition, which created probability, be wrong when studied by its offspring? What matters is that having seen one red face, all we know (and can reasonably know) is that the second face could be either red or green. Knowing the correct probability tells us something about our long-term prospects of finding another red face *assuming that the uniformity decried by the authors is imposed on the sample space*, but nothing about what we are likely to find once we turn the card^3^.

## Honesty is the Best Policy?

Statistics anxiety is a ubiquitous feature of social science courses. Part of the blame lies with the lack of practice, reputation of statistics as a “difficult” subject and mathematics-related issues. At the same time, learning to think statistically creates a conflict between intuition and the objective framework that constantly falsifies and challenges our understanding of how the world works. Although this is not necessarily wrong in itself, a closer inspection of probabilistic thinking shows that the counterintuitive nature of statistics does not originate in some deeper truth inaccessible to a lay observer, but is an unavoidable consequence of the dissonance between the fundamental limitations of human cognition and attempts to overcome these by means of mathematical formalism.

After years of training, some students conquer their anxiety and become proficient. As recommended in the literature, when facing the next generation of students, the newly fledged expert has to present a confident front and readily offer answers to difficult questions. But how can they maintain their confidence in the light of the finding that a large majority of expert researchers are well-nigh incompetent? In addition to focusing on putative antecedents of statistics anxiety, experts need to start a dialog that will shift the focus from the viability of various testing methods (e.g., the null-hypothesis significance testing, NHST) to discussing the appropriate role of statistics in research and more generally, science.

The first step could be to acknowledge the fundamental limitations of human mind and place statistics in this context. Rather than a panacea capable of advancing knowledge, probability and statistics should be viewed as an attempt to extend our informational reach into domains that are inherently beyond our grasp. We cannot know how successful our efforts are because the available tools are too simple to provide a complete (or even a partial) description of a phenomenon under observation. While being honest about limitations of statistics might not endear the lecturer to students who often crave certainty, honesty might pay off in the long run in terms of managing anxiety and unrealistic expectations as well as reducing the appeal of questionable practices. For if the relationship between statistics and reality is understood, more attention might be devoted to the psychological importance of experiments and less to the statistical significance of the result. At the same time, such a conceptual shift must be preceded by a substantial expert debate leading to a new consensus.

## Conclusion

The ubiquitous problem of statistics anxiety has been investigated from many angles including gender (Rodarte-Luna and Sherry, 2008), motivation (Lavasani et al., 2014), and personality (Chew and Dillon, 2014b). However, none of the studies has considered that discomfort could partly originate in the disconnect between the certainty with which statistics is taught and the fundamental uncertainty inherent in it. This is of particular importance for psychologists who are expected to show a deeper understanding of the relationship between the mind and the statistical apparatus used to investigate it. In conclusion, I would like to offer the following summary which might reassure students next time they think they are incompetent because they do not understand probability and statistics:

a. Probability is an attempt to control uncertainty. It has no “laws”; it does not generate new information and has no predictive power. Ability to manipulate probabilities mathematically has no impact on individual outcomes of real-life processes. Predictability depends on the complexity of the phenomenon under observation and available resources. The more we know about the process, the more we know about its outcomes.
b. Statistics can help with extracting information from noise, but the trade-off is the increase in uncertainty with respect to interpretation. Rather than allowing us to “gain one up on the Universe,” statistics is subject to the same fundamental cognitive limitations that necessitated its birth.
c. Probability models serve as *ad hoc* aids in framing research and providing reassurance and not as guarantors of truth. Outcomes of experiments might or might not come from a particular stochastic process but this can never be confirmed (or falsified). If someone objects that this does not affect the validity of statistical inference, the confused student would be justified in wondering why statistical models are used at all. The final arbiters of veridicality of a result are effect size (Cohen, 1969) and reproducibility^4^. These factors however say nothing about its importance.
d. Randomness is a mathematical idealization of subjective complexity. Students should be made aware that there is no such thing as a “random” process and that they are not in error when failing to reason or behave in accordance with probabilistic models. The problem lies elsewhere.

### Conflict of Interest Statement

The author declares that the research was conducted in the absence of any commercial or financial relationships that could be construed as a potential conflict of interest.

## Footnotes

1 The fundamental disconnect between requirements of real-life research and randomness has caused a gradual weakening of the strict definition of the latter. Thus, Shannon (1948) speaks of a “known” random source and some authors have attempted to analyze the structure of random processes (e.g., Sun and Wang, 2010). Such attempts at “taming” randomness simply confirm the fundamental incompatibility between abstract probabilistic concepts and human perception and cognition (Aksentijevic, 2015).

2 The sample consisted of over a 1000 students from an elite university.

3 One of the greatest mathematicians of the twentieth century, Paul Erdös refused to accept the correct solution to the related “Monty Hall” problem (Vazsonyi, 1999). The solution depends on all prescribed possibilities being available equally often. This presumes uniformity—which is viewed as a fallacy when applied to individual outcomes. Also, see Keynes (1921, Chapter 5) on the impossibility of adjudging the truth of these alternative interpretations.

4 Even these are ultimately subjective. According to Cohen’s (1969) convention, large effects are visible to the naked eye (visible difference). Reproducibility is another macroscopic subjective criterion (visible similarity).
